# Development, deployment, and feature interpretability of a three-class prediction model for pulmonary diseases

**DOI:** 10.1186/s13244-025-02020-7

**Published:** 2025-06-26

**Authors:** Zhenyu Cao, Gang Xu, Yuan Gao, Jianying Xu, Fengjuan Tian, Hengfeng Shi, Dengfa Yang, Zongyu Xie, Jian Wang

**Affiliations:** 1 Department of Radiology, Tongde Hospital of Zhejiang Province Afflicted to Zhejiang Chinese Medical University (Tongde Hospital of Zhejiang Province), Hangzhou, China; 2 Department of Radiology, Xin Hua Hospital of Huainan, Huainan, China; 3 Department of Radiology, Sir Run Run Shaw Hospital, Zhejiang University School of Medicine, Hangzhou, China; 4 Department of Radiology, Anqing Municipal Hospital, Anqing, China; 5 Department of Radiology, Taizhou Municipal Hospital, Taizhou, China; 6 Department of Radiology, The First Affiliated Hospital of Bengbu Medical University, Bengbu, China

**Keywords:** Machine learning, SHAP, Rimmed sign, Satellite lesion

## Abstract

**Purpose:**

To develop a high-performance machine learning model for predicting and interpreting features of pulmonary diseases.

**Patients and methods:**

This retrospective study analyzed clinical and imaging data from patients with non-small cell lung cancer (NSCLC), granulomatous inflammation, and benign tumors, collected across multiple centers from January 2015 to October 2023. Data from two hospitals in Anhui Province were split into a development set (*n* = 1696) and a test set (*n* = 424) in an 8:2 ratio, with an external validation set (*n* = 909) from Zhejiang Province. Features with *p* < 0.05 from univariate analyses were selected using the Boruta algorithm for input into Random Forest (RF) and XGBoost models. Model efficacy was assessed using receiver operating characteristic (ROC) analysis.

**Results:**

A total of 3030 patients were included: 2269 with NSCLC, 529 with granulomatous inflammation, and 232 with benign tumors. The Obuchowski indices for RF and XGBoost in the test set were 0.7193 (95% CI: 0.6567–0.7812) and 0.8282 (95% CI: 0.7883–0.8650), respectively. In the external validation set, indices were 0.7932 (95% CI: 0.7572–0.8250) for RF and 0.8074 (95% CI: 0.7740–0.8387) for XGBoost. XGBoost achieved better accuracy in both the test (0.81) and external validation (0.79) sets. Calibration Curve and Decision Curve Analysis (DCA) showed XGBoost offered higher net clinical benefit.

**Conclusion:**

The XGBoost model outperforms RF in the three-class classification of lung diseases.

**Critical relevance statement:**

XGBoost surpasses Random Forest in accurately classifying NSCLC, granulomatous inflammation, and benign tumors, offering superior clinical utility via multicenter data.

**Key Points:**

- Lung cancer classification model has broad clinical applicability.
- XGBoost outperforms random forests using CT imaging data.
- XGBoost model can be deployed on a website for clinicians.

**Graphical Abstract:**

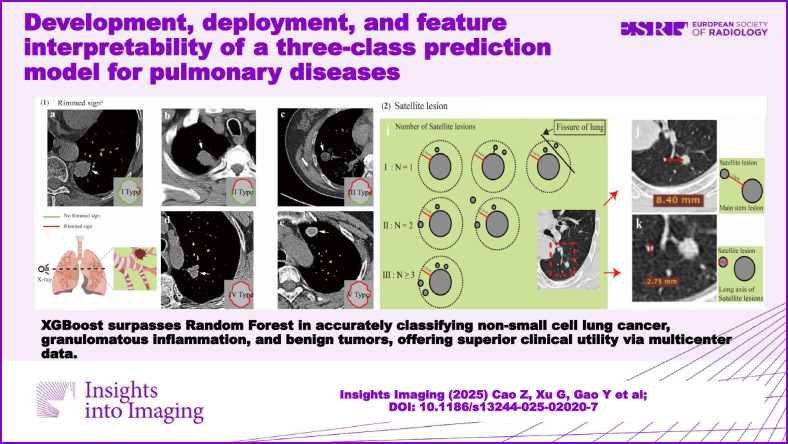

## Introduction

Non-small cell lung cancer (NSCLC) is a common subtype of lung cancer, accounting for 80% of all lung cancer cases in China and 85% of all malignant lung tumors globally [1–3]. In recent years, the mortality rate of NSCLC has decreased owing to the widespread use of lung screening, which has made early detection of lung tumors possible. However, there are challenges; some benign lung tumors and granulomatous inflammation are misdiagnosed as NSCLC [4]. Owing to the limited specificity of CT, these patients undergo excessive treatments and examinations, leading to unnecessary waste of medical resources. Therefore, developing an efficient and accurate lung disease prediction model is urgently needed.

Machine learning (ML) technologies enable researchers to learn from historical instances and build complex patterns from large, noisy, or complex datasets [5]. ML methods are now widely used in disease detection and classification, providing insights and conclusions that standard statistical procedures cannot achieve [6, 7]. In this study, we input various features into random forest (RF) and extreme gradient boosting (XGBoost) models to develop classification models for distinguishing non-small cell lung cancer, pulmonary granulomatous inflammation, and benign lung tumors. Although many studies have described the features of these diseases in CT images, reports on lung disease features in terms of border signs and satellite nodules are still limited, and few studies have utilized machine learning techniques to identify these three types of tumors. Our research included a substantial sample size of patients with non-small cell lung cancer, pulmonary granulomatous inflammation, and benign lung tumors. We aim to analyze these diseases via machine learning and compare their diagnostic performance to achieve effective differentiation and feature interpretation of these conditions [8].

Among the standard lung imaging features, the rimmed sign refers to a circle of high-density imaging around a lung mass, which may reflect the inflammatory reaction of the tumor microenvironment or the characteristics of the invasive growth of the tumor. Satellite lesions are small, scattered foci found around the main lesion and are associated with the development of pneumonia and local spread [9]. These two imaging features have shown potential value in distinguishing benign, malignant, and inflammatory lung lesions, as well as in assessing tumor invasiveness and predicting patient prognosis [10].

This study aimed to create a prediction model for lung masses by combining the rimmed sign and satellite lesions. By analyzing clinical data and imaging features, we aimed to improve the accuracy of diagnosing malignant pulmonary lesions, benign pulmonary lesions, and pneumonia. This study will help improve the differential diagnosis of these lung diseases.

## Materials and methods

### Patients

This study constitutes a retrospective analysis conducted across multiple centers in Anhui and Zhejiang Provinces, with ethical approval granted under the following reference numbers: LWSL202300145 (The First Affiliated Hospital of Bengbu Medical University), 83230471 (Anqing Municipal Hospital), 2022029-JY (Tongde Hospital) and 2023439 (Taizhou Municipal Hospital). Notably, informed consent was waived for all participating patients.

**Fig. 1:**
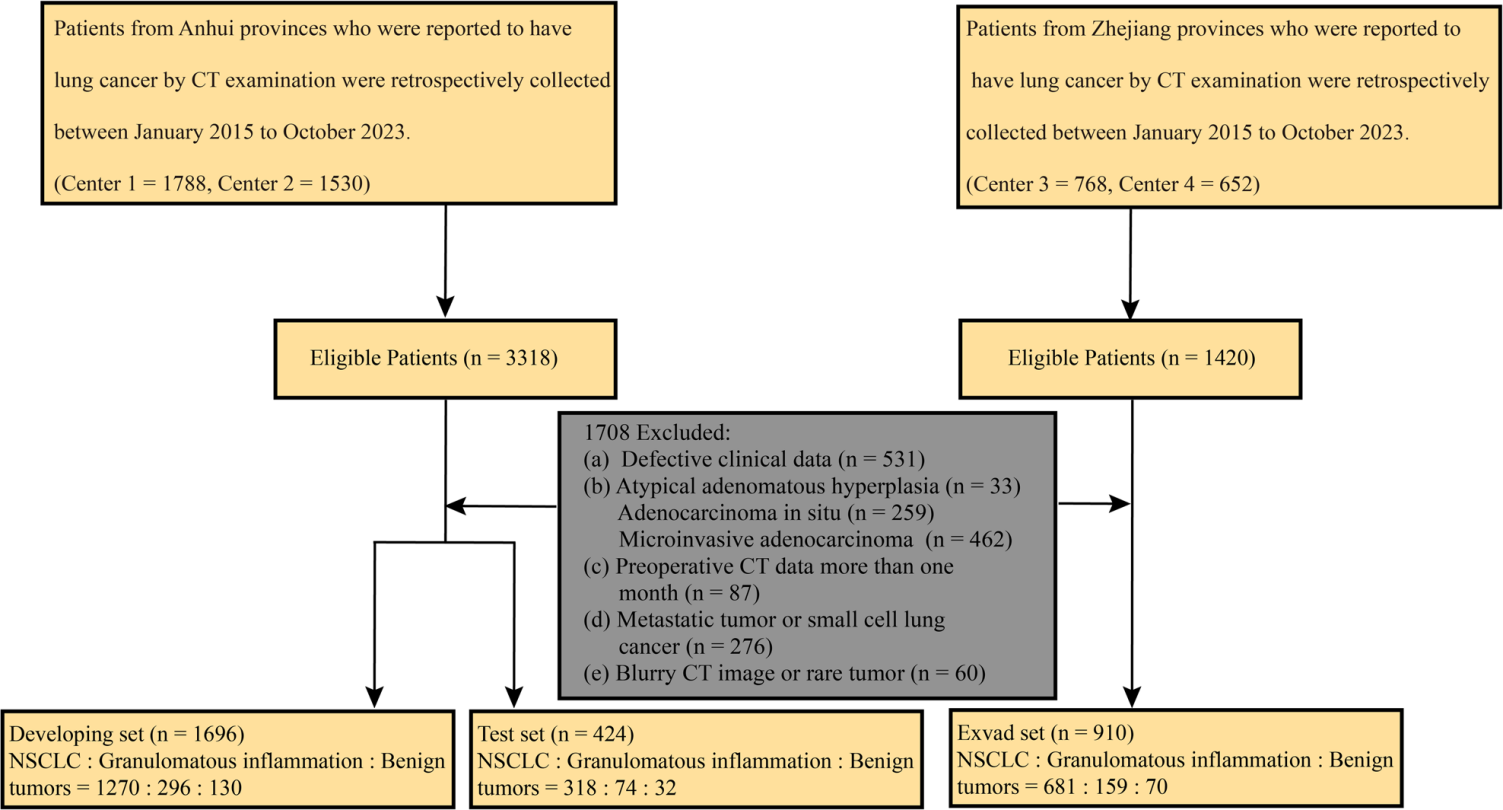
Patient flow chart. NSCLC, non-small cell lung cancer

This study retrospectively enrolled patients with CT-detected pulmonary nodules or masses scheduled for surgical resection between January 2015 and October 2023. All patients underwent preoperative thin-section CT (slice thickness ≤ 2 mm). Definitive postoperative pathological diagnoses categorized lesions as non-small cell lung cancer, benign tumors, or granulomatous inflammation. A total of 4738 patients met the inclusion criteria (Fig. 1). The exclusion criteria were patients with incomplete clinical data or those diagnosed with atypical adenomatous hyperplasia, adenocarcinoma in situ, or microinvasive adenocarcinoma. Additionally, patients with preoperative CT scans older than 1 month, those diagnosed with metastatic tumors or small cell lung cancer, and those with low-quality CT images or rare tumor types were excluded from the study. A total of 3030 patients were included in the final analysis. It includes detailed information on patient demographics, radiographic imaging data, pathological examinations, and biochemical tests.

### CT image achievement

Chest CT imaging was conducted via various CT scanners, including the GE Optima CT680, GE Optima CT540, GE Bright Speed 16, SIEMENS SOMATOM Definition Flash CT, and Philips Incisive CT. The tube voltage was set at 120 kV, and the tube current was automatically regulated, ranging from 150–300 mA. The slice thickness and spacing were both 5 mm. Thin-slice reconstruction was performed via the standard reconstruction algorithm with a slice thickness and spacing ranging from 0.5 to 1.25 mm. All radiological assessments were conducted exclusively on non-contrast CT images. For patients who underwent contrast-enhanced CT scans, only the non-contrast components of the scans were used for feature evaluation to ensure consistency and generalizability across the cohort.

### Image analysis

**Fig. 2:**
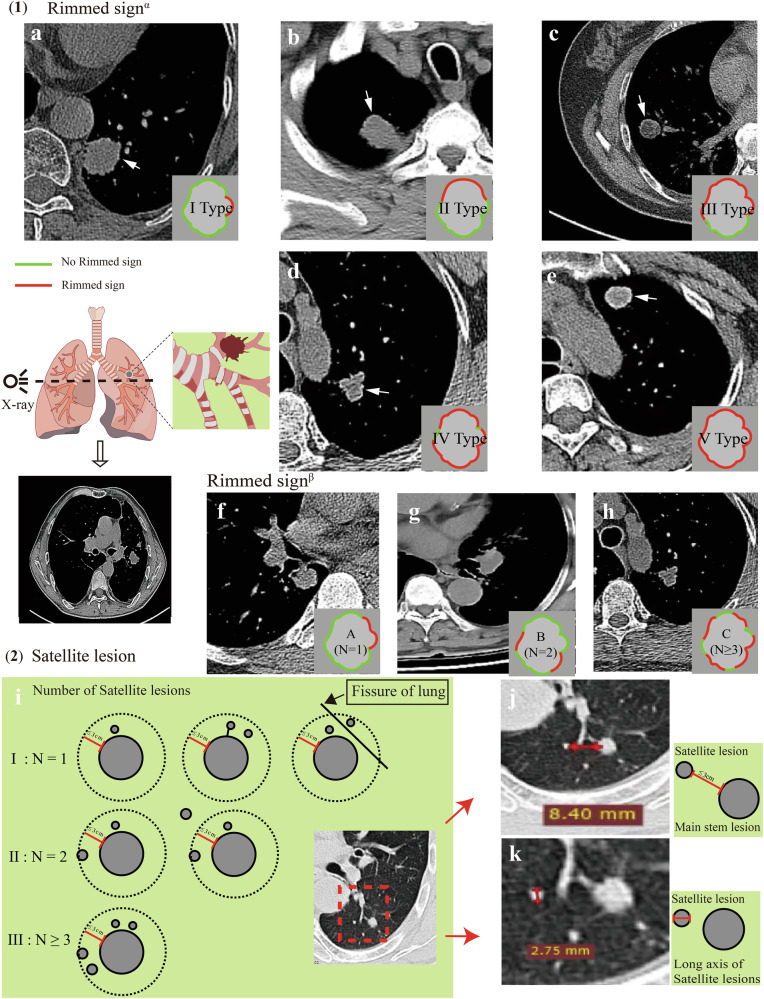
Definition of the rimmed sign and satellite lesion. (**1**) Rimmed sign: There was a high-density signal at the edge of the mass. rimmed sign^α^: I: The length is less than 25% (**a**), II: The length is between 25 and 50% (**b**), III: The length is between 50 and 75% (**c**), IV: The length is between 75 and 100% (**d**), V: The length is equal to 100% (**e**). rimmed sign^β^: A: A persistent high-density line is observed in the rimmed sign (**f**). B: Two persistent high-density lines are observed in the rimmed sign (**g**). C: More than three persistent high-density lines are observed in the rimmed sign (**h**). (**2**) Satellite lesion: Small lesions were present within a 3-cm radius of the primary lesion. Number of satellite lesions: I: Only one small lesion was present within a 3-cm radius of the primary lesion, and it did not exhibit a connection to the primary stem lesion, nor did it traverse the fissure of the lung. II: Two small lesions were present within a 3-cm radius of the primary lesion. III: More than three small lesions were present within a 3-cm radius of the primary lesion (**i**). Distance between the satellite lesion and the main stem lesion, and it did not cross the lung lobes: the distance shown in this case was 8.40 mm (**j**). Long axis of satellite lesions: The distance shown in this case was 2.75 mm (**k**)

Clinical data, including sex, age, smoking status, operation status, underlying diseases, tumor markers, and pulmonary diseases, were obtained from the patients’ medical records. Two certified radiologists (with 5 and 15 years of experience) evaluated the chest CT images via a unified standard. CT data were analyzed for the following variables: lesion shape (regular or irregular), location, mGGO, margin (with or without spiculation), air space, air bronchogram, pleural tags, calcification, pleural effusion, halo sign, cut sign, reverse halo sign, long diameter (LD), short diameter (SD), maximum CT attenuation (CTmax), minimum CT attenuation (CTmin), mean CT attenuation (CTmean), and standard deviation of CT attenuation (CTsd) [11–13]. The following imaging features were also recorded: the rimmed sign was defined as a high-density signal at the edge of the mass. The rimmed sign α was defined as the proportion of high-density signals at the edge of the mass to the total edge. rimmed signβ was defined as the number of high-density signals at the edge of the mass. Satellite lesions were defined as small lesions within a 3-cm radius of the primary lesion. We also recorded the largest measurement diameter among the satellite lesions, the distance between the satellite lesion and the main stem lesion, which does not cross the lobes, and the number of satellite lesions (Fig. 2). Radiologists do not refer to radiology reports when evaluating CT features. To assess diagnostic consistency, intra-class correlation coefficients (ICC) were used for continuous or ordinal measurements, with ICC > 0.75 defined as good agreement. For categorical variables, Kappa (k) was employed, where k > 0.8 indicated substantial inter-rater reliability. Both statistical methods ensured a comprehensive evaluation of diagnostic consistency across different data types.

### Statistical analysis

Statistical analysis: Univariate analysis was performed via SPSS 26, and machine learning classification predictions were made via Python (3.9.19). Model Construction: Among the 3030 CT scans, three centers in Anhui Province were divided into a developing set (1696 cases) and a test set (424 cases) in an 8:2 ratio, while one center in Zhejiang Province provided 910 cases for the Exvad set. Pathological results were used as the diagnostic criterion. In the models, granulomatous inflammation was coded as Class 1, benign tumors as Class 2, and non-small cell lung cancer as Class 3. Classifier 1 was used to distinguish granulomatous inflammation from nongranulomatous inflammation, Classifier 2 was used to differentiate benign tumors from nonbenign tumors, and Classifier 3 was used to distinguish non-small cell lung cancer from both benign tumors and granulomatous inflammation. Univariate analysis was conducted to evaluate features, using the chi-square test for categorical variables and one-way ANOVA for continuous variables. Statistically significant features were selected using the Boruta algorithm, executed 20 times to ensure stability, based on random forest, and Recursive Feature Elimination (RFE) was combined with 5-fold cross-validation to identify key risk factors. The chosen features served as input variables for constructing models utilizing the random forest (RF) and extreme gradient boosting (XGBoost) machine learning algorithms. The hyperparameters of these models were optimized via Bayesian optimization. Model performance was subsequently assessed via receiver operating characteristic (ROC) curves, calibration curves, and clinical decision analysis (DCA) [14–16]. Shapley Additive Explanations (SHAP) and Individual Conditional Expectation (ICE) plots were used to interpret the influence of selected features on the prediction of pathological outcomes, providing visual insights into how each feature affected the model’s predictions at both individual and global levels. The random forest and XGBoost Python implementation is available at https://github.com/Xiaohuanghua66/Rimmed-sign-and-Satellite-lesion/tree/main.

## Results

### Clinical characteristics and CT findings

A total of 3030 patients were included in the study, comprising 1383 males and 1647 females. The cohort was divided into three groups: 2269 patients with non-small cell lung cancer (NSCLC), 529 with granulomatous inflammation, and 232 with benign tumors, with a mean age of 59.6 ± 11.2 years. Table 1 provides a detailed overview of the demographic characteristics of the study participants. The distribution of patients from two independent centers into the development and test sets is as follows: The First Affiliated Hospital of Bengbu Medical College in Anhui Province contributed 1029 patients, while Anqing Municipal Hospital in Anhui Province contributed 1092 patients. This distribution includes 1588 NSCLC patients, 370 granulomatous inflammation patients, and 162 benign tumor patients. Additionally, 481 patients from Tongde Hospital and 429 patients from Taizhou Municipal Hospital in Zhejiang Province were allocated to the external validation set, comprising 681 NSCLC patients, 159 granulomatous inflammation patients, and 70 benign tumor patients. Among the variables analyzed, only four—sex, tumor indicator, calcification, and reverse halo sign—did not demonstrate statistically significant differences between patients from the two provinces, whereas all other features showed significant differences (*p* < 0.05) (Table S1).

**Table 1:** Clinical characteristics of the study sample stratified by three pathological types

Characteristics	Total number of patients									*p*-value (developing set)
	Non-small cell lung cancer (*n* = 2269)			Granulomatous inflammation (*n* = 529)			Benign tumors (*n* = 232)			
	Developing set (*n* = 1270)	Test set (*n* = 318)	Exvad set (*n* = 681)	Developing set (*n* = 296)	Test set (*n* = 74)	Exvad set (*n* = 159)	Developing set (*n* = 130)	Test set (*n* = 32)	Exvad set (*n* = 70)	
Sex										**< 0.001**
Female	730 (57.48)	191 (60.06)	354 (51.98)	128 (43.24)	28 (37.84)	65 (40.88)	76 (58.46)	22 (68.75)	53 (75.71)	
Male	540 (42.52)	127 (39.94)	327 (48.02)	168 (56.76)	46 (62.16)	94 (59.12)	54 (41.54)	10 (31.25)	17 (24.29)	
Age^#^	59.74 ± 10.94	61.61 ± 9.80	62.79 ± 10.44	57.33 ± 11.50	59.42 ± 9.90	52.75 ± 12.94	55.53 ± 10.57	52.25 ± 13.07	54.14 ± 12.50	0.570
Smoke										**0.004**
Never	1101 (86.69)	260 (81.76)	534 (78.41)	250 (84.46)	55 (74.32)	131 (82.39)	126 (96.92)	28 (87.50)	61 (87.14)	
Current	109 (8.58)	40 (12.58)	83 (12.19)	28 (9.46)	15 (20.27)	21 (13.21)	3 (2.31)	1 (3.12)	8 (11.43)	
Former	60 (4.72)	18 (5.66)	64 (9.40)	18 (6.08)	4 (5.41)	7 (4.40)	1 (0.77)	3 (9.38)	1 (1.43)	
Surgical history										**0.019**
No	1120 (88.19)	263 (82.70)	590 (86.64)	277 (93.58)	64 (86.49)	143 (89.94)	119 (91.54)	30 (93.75)	62 (88.57)	
Lung cancer	14 (1.10)	28 (8.81)	52 (7.64)	3 (1.01)	3 (4.05)	2 (1.26)	0 (0.00)	0 (0.00)	1 (1.43)	
Digestive tract cancer	28 (2.20)	4 (1.26)	12 (1.76)	6 (2.03)	2 (2.70)	4 (2.52)	1 (0.77)	0 (0.00)	0 (0.00)	
Other cancer	108 (8.50)	23 (7.23)	27 (3.96)	10 (3.38)	5 (6.76)	10 (6.29)	10 (7.69)	2 (6.25)	7 (10.00)	
HBP										**< 0.001**
Yes	20 (1.57)	5 (1.57)	176 (25.84)	3 (2.31)	18 (24.32)	39 (24.53)	8 (2.70)	5 (15.62)	14 (20.00)	
DM										**< 0.001**
Yes	14 (1.10)	0 (0.00)	61(8.96)	4 (1.35)	15 (20.27)	27 (16.98)	1 (0.77)	2 (6.25)	2 (2.86)	
Emphysema or Bullae of lung										0.063
Yes	188 (14.80)	54 (16.98)	181 (26.58)	54 (18.24)	12 (16.22)	14 (8.81)	15 (11.54)	1 (3.12)	10 (14.29)	
HVP										0.548
Yes	174 (13.70)	37 (11.64)	75 (11.01)	44 (14.86)	10 (13.51)	15 (9.43)	13 (10.00)	5 (15.62)	5 (7.14)	
ID										0.657
Yes	6 (0.47)	2 (0.63)	30 (4.41)	2 (0.68)	0 (0.00)	1 (0.63)	0 (0.00)	0 (0.00)	0 (0.00)	
Bronchiectasis										0.502
Yes	11 (0.87)	1 (0.31)	15 (2.20)	2 (0.68)	0 (0.00)	2 (1.26)	0 (0.00)	0 (0.00)	0 (0.00)	
MLC										0.241
Yes	35 (2.76)	9 (2.83)	37 (5.43)	11 (3.72)	0 (0.00)	5 (3.14)	1 (0.77)	0 (0.00)	1 (1.43)	
Tumor indicator										**< 0.001**
Yes	382 (30.13)	68 (21.38)	229 (33.63)	57 (19.26)	10 (13.51)	18 (11.32)	21 (16.15)	3 (9.38)	7 (10.00)	
mGGO										**< 0.001**
0	340 (26.77)	112 (35.22)	95 (13.95)	35 (11.82)	6 (8.11)	1 (0.63)	4 (3.08)	0 (0.00)	0 (0.00)	
≤ 25%	415 (32.68)	65 (20.44)	123 (18.06)	28 (9.46)	9 (12.16)	3 (1.89)	3 (2.31)	2 (6.25)	1 (1.43)	
≤ 50%	107 (8.43)	17 (5.35)	36 (5.29)	8 (2.70)	1 (1.35)	4 (2.52)	1 (0.77)	0 (0.00)	0 (0.00)	
≤ 75%	98 (7.72)	21 (6.60)	51 (7.49)	6 (2.03)	1 (1.35)	3 (1.89)	1 (0.77)	0 (0.00)	0 (0.00)	
< 100%	123 (9.69)	19 (5.97)	62 (9.10)	13 (4.39)	2 (2.70)	3 (1.89)	4 (3.08)	0 (0.00)	2 (2.86)	
100%	187 (14.72)	84 (26.42)	314 (46.11)	206 (69.59)	55 (74.32)	145 (91.19)	117 (90.00)	30 (93.75)	67 (95.71)	
Location										**0.001**
Right upper lobe	400 (31.50)	123 (38.68)	202 (29.66)	95 (32.09)	28 (37.84)	42 (26.42)	28 (21.54)	3 (9.38)	9 (12.86)	
Right middle lobe	90 (7.09)	23 (7.23)	46 (6.75)	35 (11.82)	10 (13.51)	9 (5.66)	16 (12.31)	3 (9.38)	9 (12.86)	
Right lower lobe	270 (21.26)	59 (18.55)	129 (18.94)	62 (20.95)	17 (22.97)	41 (25.79)	28 (21.54)	10 (31.25)	16 (22.86)	
Left upper lobe	322 (25.35)	70 (22.01)	183 (26.87)	55 (18.58)	8 (10.81)	30 (18.87)	28 (21.54)	5 (15.62)	13 (18.57)	
Left lower lobe	188 (14.80)	43 (13.52)	121 (17.77)	49 (16.55)	11 (14.86)	37 (23.27)	30 (23.08)	11 (34.38)	23 (32.86)	
Morphology										**< 0.001**
Round	28 (2.20)	20 (6.29)	27 (3.96)	21 (7.09)	11 (14.86)	27 (16.98)	71 (4.19)	9 (28.12)	36 (51.43)	
Oval	103 (8.11)	67 (21.07)	90 (13.22)	77 (26.01)	18 (24.32)	28 (17.61)	228 (13.44)	11 (34.38)	20 (28.57)	
Irregular	1139 (89.69)	231 (72.64)	564 (82.82)	198 (66.89)	45 (60.81)	104 (65.41)	1397 (82.37)	12 (37.50)	14 (20.00)	
Lobulation										**< 0.001**
Yes	943 (74.25)	149 (46.86)	479 (70.34)	90 (30.41)	24 (32.43)	81 (50.94)	32 (24.62)	8 (25.00)	10 (14.29)	
Spiculation										**< 0.001**
No	779 (61.34)	236 (74.21)	369 (54.19)	220 (74.32)	52 (70.27)	109 (68.55)	129 (99.23)	32 (100.00)	70 (100.00)	
Short (≤ 10 mm)	359 (28.27)	60 (18.87)	261 (38.33)	39 (13.18)	6 (8.11)	12 (7.55)	1 (0.77)	0 (0.00)	0 (0.00)	
Long (> 10 mm)	132 (10.39)	22 (6.92)	51 (7.49)	37 (12.50)	16 (21.62)	38 (23.90)	0 (0.00)	0 (0.00)	0 (0.00)	
Air space										**< 0.001**
0	1032 (81.26)	249 (78.30)	524 (76.95)	274 (92.57)	68 (91.89)	136 (85.53)	124 (95.38)	29 (90.62)	69 (98.57)	
≤ 5	138 (10.87)	52 (16.35)	115 (16.89)	14 (4.73)	5 (6.76)	12 (7.55)	2 (1.54)	1 (3.12)	1 (1.43)	
> 5	95 (7.48)	15 (4.72)	38 (5.58)	7 (2.36)	1 (1.35)	7 (4.40)	4 (3.08)	2 (6.25)	0 (0.00)	
cavity	5 (0.39)	2 (0.63)	4 (0.59)	1 (0.34)	0 (0.00)	4 (2.52)	0 (0.00)	0 (0.00)	0 (0.00)	
Air bronchogram										**< 0.001**
No	1003 (78.98)	264 (83.02)	503 (73.86)	268 (90.54)	62 (83.78)	113 (71.07)	128 (98.46)	31 (96.88)	68 (97.14)	
Without tracheal deformation	55 (4.33)	19 (5.97)	63 (9.25)	11 (3.72)	9 (12.16)	42 (26.42)	1 (0.77)	1 (3.12)	2 (2.86)	
Accompanied by tracheal deformation or traction displacement	146 (11.50)	29 (9.12)	63 (9.25)	11 (3.72)	1 (1.35)	3 (1.89)	0 (0.00)	0 (0.00)	0 (0.00)	
Bronchiectasis or ectopic displacement due to traction	66 (5.20)	6 (1.89)	52 (7.64)	6 (2.03)	2 (2.70)	1 (0.63)	1 (0.77)	0 (0.00)	0 (0.00)	
Pleural tags										**< 0.001**
Type 0	406 (31.97)	118 (37.11)	183 (26.87)	104 (35.14)	33 (44.59)	43 (27.04)	73 (56.15)	17 (53.12)	42 (60.00)	
Type I	566 (44.57)	121 (38.05)	227 (33.33)	104 (35.14)	25 (33.78)	72 (45.28)	52 (40.00)	13 (40.62)	28 (40.00)	
Type II	143 (11.26)	44 (13.84)	87 (12.78)	42 (14.19)	7 (9.46)	31 (19.50)	4 (3.08)	2 (6.25)	0 (0.00)	
Type III	46 (3.62)	10 (3.14)	64 (9.40)	18 (6.08)	2 (2.70)	1 (0.63)	1 (0.77)	0 (0.00)	0 (0.00)	
Type IV	109 (8.58)	25 (7.86)	120 (17.62)	28 (9.46)	7 (9.46)	12 (7.55)	0 (0.00)	0 (0.00)	0 (0.00)	
Calcification										**< 0.001**
Yes	15 (1.18)	3 (0.94)	18 (2.64)	25 (8.45)	6 (8.11)	6 (3.77)	17 (13.08)	7 (21.88)	11 (15.71)	
Pleural effusion										0.664
Yes	2 (0.16)	5 (1.57)	9 (1.32)	0 (0.00)	2 (2.70)	1 (0.63)	0 (0.00)	0 (0.00)	0 (0.00)	
Halo sign										**< 0.001**
Yes	6 (0.47)	4 (1.26)	24 (3.52)	12 (4.05)	6 (8.11)	31 (19.50)	1 (0.77)	1 (3.12)	0 (0.00)	
Cut sign										**< 0.001**
Yes	1 (0.08)	1 (0.31)	11 (1.62)	8 (2.70)	0 (0.00)	7 (4.40)	1 (0.77)	0 (0.00)	0 (0.00)	
Reverse halo sign										**0.001**
Yes	2 (0.16)	0 (0.00)	1 (0.15)	5 (1.69)	0 (0.00)	1 (0.63)	0 (0.00)	0 (0.00)	0 (0.00)	
LD (mm)^#^	19.79 ± 9.93	17.83 ± 9.85	21.79 ± 11.02	13.61 ± 8.54	16.11 ± 10.46	21.86 ± 14.27	14.94 ± 11.37	17.62 ± 11.99	17.29 ± 10.58	0.163
SD (mm)^#^	14.65 ± 7.52	13.30 ± 7.49	16.17 ± 8.23	10.01 ± 6.04	11.66 ± 6.19	16.29 ± 10.96	12.20 ± 9.64	14.53 ± 8.49	14.81 ± 9.32	**0.001**
CTmax (HU)^#^	75.79 ± 200.52	−3.01 ± 201.47	94.17 ± 160.67	120.31 ± 194.71	106.55 ± 192.70	178.06 ± 87.01	157.54 ± 133.92	173.84 ± 81.89	152.90 ± 81.88	**< 0.001**
CTmin (HU)^#^	−234.94 ± 243.51	−220.74 ± 237.10	−183.38 ± 221.91	−108.64 ± 210.88	−125.27 ± 187.17	−91.69 ± 104.61	−61.72 ± 118.03	−76.34 ± 71.64	−84.41 ± 67.10	**< 0.001**
CTmean (HU)^#^	−75.79 ± 201.42	−107.88 ± 204.44	−32.10 ± 158.52	4.14 ± 188.09	−14.80 ± 162.95	44.13 ± 50.11	48.63 ± 103.44	43.23 ± 41.67	37.52 ± 27.39	**< 0.001**
CTsd (HU)^#^	92.66 ± 52.56	66.09 ± 44.81	71.23 ± 52.71	69.52 ± 40.28	63.24 ± 55.26	68.36 ± 35.14	67.65 ± 44.25	61.51 ± 28.40	57.34 ± 22.41	**< 0.001**
Rimmed sign										**< 0.001**
No	1225 (96.46)	308 (96.86)	609 (89.43)	256 (86.49)	67 (90.54)	103 (64.78)	88 (67.69)	19 (59.38)	32 (45.71)	
Yes	45 (3.54)	10 (3.14)	72 (10.57)	40 (13.51)	7 (9.46)	56 (35.22)	42 (32.31)	13 (40.62)	38 (54.29)	
Rimmed sign^α^										**< 0.001**
L = 0%	1225 (96.46)	308 (96.86)	610 (89.57)	256 (86.49)	67 (90.54)	103 (64.78)	88 (67.69)	19 (59.38)	32 (45.71)	
L < 25%	2 (0.16)	1 (0.31)	11 (1.62)	2 (0.68)	0 (0.00)	1 (0.63)	0 (0.00)	0 (0.00)	0 (0.00)	
25% ≤ L < 50%	4 (0.31)	2 (0.63)	20 (2.94)	3 (1.01)	1 (1.35)	6 (3.77)	0 (0.00)	0 (0.00)	1 (1.43)	
50% ≤ L < 75%	9 (0.71)	6 (1.89)	21 (3.08)	7 (2.36)	2 (2.70)	5 (3.14)	3 (2.31)	0 (0.00)	4 (5.71)	
75% ≤ L < 100%	14 (1.10)	1 (0.31)	16 (2.35)	12 (4.05)	2 (2.70)	21 (13.21)	11 (8.46)	3 (9.38)	10 (14.29)	
L = 100%	16 (1.26)	0 (0.00)	3 (0.44)	16 (5.41)	2 (2.70)	23 (14.47)	28 (21.54)	10 (31.25)	23 (32.86)	
Rimmed sign^β^										**< 0.001**
*N* = 0	1225 (96.46)	308 (96.86)	610 (89.57)	256 (86.49)	67 (90.54)	103 (64.78)	88 (67.69)	19 (59.38)	32 (45.71)	
*N* = 1	27 (2.13)	2 (0.63)	17 (2.50)	31 (10.47)	2 (2.70)	35 (22.01)	36 (27.69)	11 (34.38)	26 (37.14)	
*N* = 2	8 (0.63)	2 (0.63)	14 (2.06)	5 (1.69)	3 (4.05)	13 (8.18)	2 (1.54)	1 (3.12)	8 (11.43)	
*N* ≥ 3	10 (0.79)	6 (1.89)	40 (5.87)	4 (1.35)	2 (2.70)	8 (5.03)	4 (3.08)	1 (3.12)	4 (5.71)	
Satellite lesion										**< 0.001**
No	1268 (99.84)	314 (98.74)	663 (97.36)	264 (89.19)	61 (82.43)	102 (64.15)	130 (100.00)	32 (100.00)	67 (95.71)	
Yes	2 (0.16)	4 (1.26)	18 (2.64)	32 (10.81)	13 (17.57)	57 (35.85)	0 (0.00)	0 (0.00)	3 (4.29)	
Long axis of satellite lesion (mm)	0.01 ± 0.29	0.05 ± 0.50	0.14 ± 1.04	0.48 ± 1.94	0.81 ± 2.32	2.04 ± 3.73	0.00 ± 0.00	0.00 ± 0.00	0.10 ± 0.54	**< 0.001**
Distance between satellite lesion and main stem lesion (mm)	0.02 ± 0.50	0.08 ± 0.83	0.23 ± 1.58	0.80 ± 2.81	1.22 ± 3.84	2.58 ± 4.56	0.00 ± 0.00	0.00 ± 0.00	0.10 ± 0.49	**< 0.001**
Number of satellite lesion										**< 0.001**
*N* = 0	1268 (99.84)	314 (98.74)	663 (97.36)	264 (89.19)	61 (82.43)	102 (64.15)	130 (100.00)	32 (100.00)	67 (95.71)	
*N* = 1	1 (0.08)	3 (0.94)	14 (2.06)	5 (1.69)	4 (5.41)	20 (12.58)	0 (0.00)	0 (0.00)	2 (2.86)	
*N* = 2	0 (0.00)	0 (0.00)	1 (0.15)	10 (3.38)	0 (0.00)	2 (1.26)	0 (0.00)	0 (0.00)	1 (1.43)	
*N* ≥ 3	1 (0.08)	1 (0.31)	3 (0.44)	17 (5.74)	9 (12.16)	35 (22.01)	0 (0.00)	0 (0.00)	0 (0.00)	

The data are qualitative variables; the number of patients is located outside the brackets, the percentages are located inside the brackets, and the statistical values are Pearson’s χ^2^ test. *p*-values written in bold indicate a significant difference between lesions. Normally distributed data are expressed as the mean ± standard deviation

*HBP* high blood pressure, *DM* diabetes mellitus, *HVP* heterogeneous ventilation or perfusion, *ID* interstitial lung disease, *MLC* multiple lung comorbidity, *LD* long diameter, *SD* short diameter, *CT* X-ray computed tomography, *Rimmed sign*^*α*^ length of rimmed sign, *Rimmed sign*^*β*^ number of limbs in the rimmed sign

# The data are presented as the means ± standard deviations, and the statistical values are the results of one-way ANOVA

### Univariate analysis

Univariate analysis revealed that the *p*-values for sex, smoking status, surgical history, high blood pleasure (HBP), diabetes mellitus (DM), multiple lung comorbidity (MLC), tumor indicator, mGGO, location, morphology, lobulation, spiculation, air space, air bronchogram, pleural tags, calcification, halo sign, cut sign, reverse halo sign, SD, CTmax, CTmin, CTmean, CTsd, Rimmed sign, Rimmed sign α, Rimmed sign β, satellite lesion, long axis of satellite lesion, distance between satellite lesion and main stem lesion, and number of satellite lesions were less than 0.05. Therefore, these 31 features were included in the machine learning model (Table 1).

### Boruta analysis

**Fig. 3:**
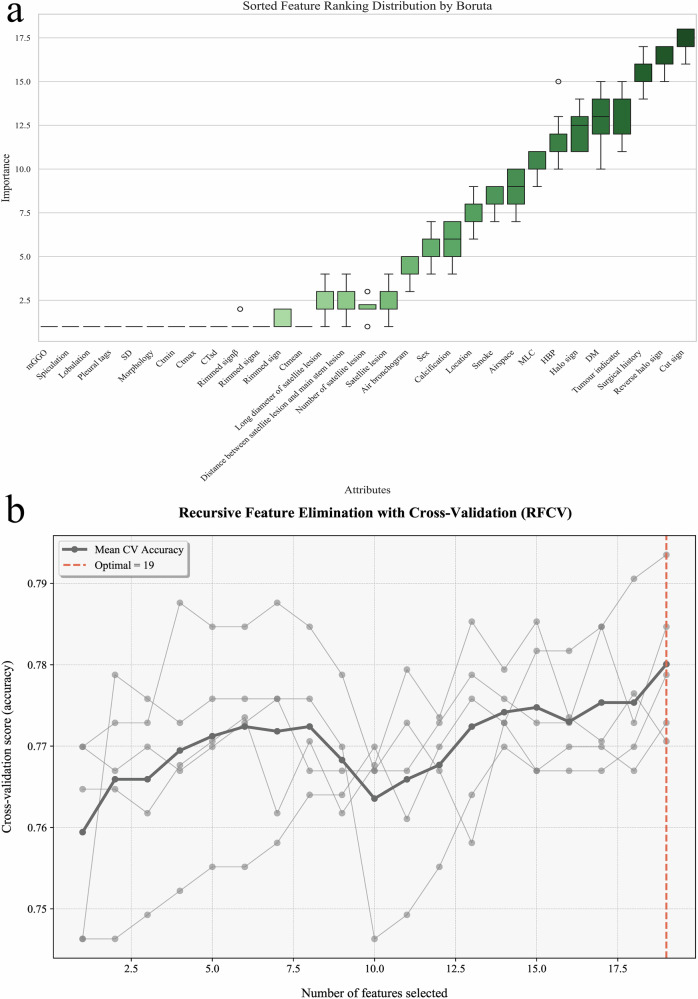
Sorted feature ranking distribution by Boruta (**a**). DM, diabetes mellitus; MLC, multiple lung comorbidity. Through recursive feature elimination (RFE) for feature selection, the highest accuracy was achieved with 19 features (gray represents five-fold cross-validation) (**b**)

Nineteen features were identified as risk factors through the Boruta algorithm on the basis of their importance rankings derived from random forest analysis. These features include the cut sign, reverse halo sign, surgical history, tumor indicator, diabetes mellitus (DM), halo sign, high blood pressure (HBP), multiple lung comorbidity (MLC), smoking status, air space, lesion location, sex, calcification, air bronchogram, satellite lesion, number of satellite lesions, distance between satellite lesions and the main stem lesion, long axis of satellite lesions, and rimmed sign (Fig. 3a). To enhance the selection of pertinent features, Recursive Feature Elimination (RFE) was utilized. RFE methodically eliminated less significant features to improve the model’s performance. This process was integrated with 5-fold cross-validation to ensure a robust evaluation of the model. Accuracy was selected as the primary metric for assessing model performance, and the findings indicated that the inclusion of 19 features resulted in the highest accuracy. This validation confirms that these features are highly predictive and significantly contribute to the model’s classification capability (Fig. 3b). Subsequently, these features were incorporated into machine learning models (Random Forest and XGBoost).

### Model comparison

**Fig. 4:**
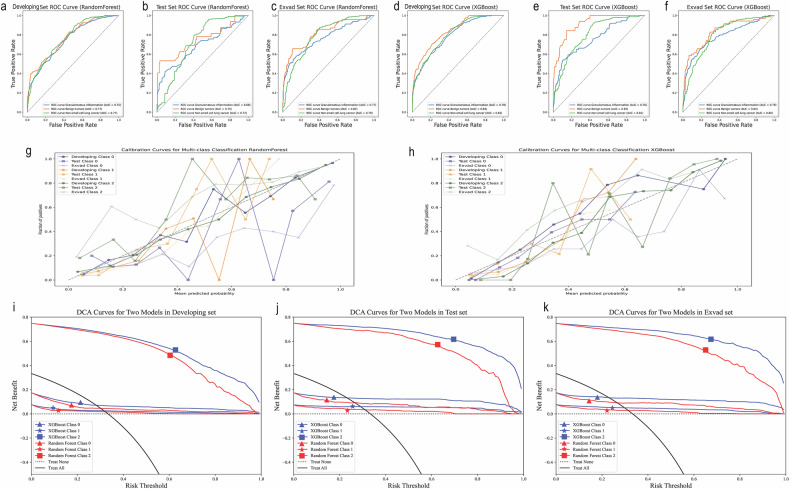
The ROC curves, calibration curves, and clinical decision curve (DCA). **a**–**f** ROC curves for the random forest and XGBoost models in both the developing and test sets. **g**, **h** Calibration curves of the Random Forest model and XGBoost model, respectively. **i**–**k** The clinical decision curve (DCA) for the developing set, test set, and Exvad set

After evaluating five machine learning algorithms (with default parameters) across six sampling techniques (random under-sampling, random over-sampling, SMOTE, Borderline-SMOTE, SVMSMOTE, and no sampling), we selected Random Forest and XGBoost for final comparison based on their averaged performance metrics (Tables S3–S5). In the developing set, the random forest model achieved AUC values of 0.75, 0.77, and 0.77 for classes 0, 1, and 2, respectively (Fig. 4a), with an Obuchowski index of 0.7599 (95% CI: 0.7361–0.7838), and an accuracy of 0.79. For the XGBoost model, the AUC values were 0.79, 0.84, and 0.81 for classes 0, 1, and 2, respectively (Fig. 4d), with an Obuchowski index of 0.8110 (95% CI: 0.7897–0.8303), and an accuracy of 0.81. The Delong test comparing the AUCs between the two models in the developing set showed a *p*-value of 0.0019 and a Z score of −3.104, indicating a statistically significant difference in model performance (Table 2).

**Table 2:** Performance comparison of the random forest and XGBoost methods using macro average and weighted average metrics

		RandomForest				XGBoost				
Dataset	Class		Precision	Recall	F1 score		Precision	Recall	F1 score	Support
Developing set	0 (Granulomatous inflammation)		0.71	0.23	0.35		0.8	0.27	0.4	296
	1 (Benign tumors)		0.51	0.29	0.37		0.59	0.33	0.42	130
	2 (Non-small cell lung cancer)		0.81	0.98	0.89		0.82	0.98	0.89	1270
	Macro average		0.68	0.5	0.54		0.73	0.53	0.57	1696
	Weighted average		0.77	0.79	0.75		0.8	0.81	0.77	1696
Test set	0 (Granulomatous inflammation)		0.56	0.31	0.4		0.65	0.3	0.41	74
	1 (Benign tumors)		0.62	0.41	0.49		0.8	0.38	0.51	32
	2 (Non-small cell lung cancer)		0.84	0.95	0.89		0.83	0.98	0.9	318
	Macro average		0.67	0.56	0.59		0.76	0.55	0.61	424
	Weighted average		0.77	0.8	0.78		0.8	0.81	0.78	424
Exvad set	0 (Granulomatous inflammation)		0.54	0.5	0.52		0.54	0.47	0.5	159
	1 (Benign tumors)		0.49	0.47	0.48		0.52	0.46	0.48	70
	2 (Non-small cell lung cancer)		0.86	0.87	0.87		0.86	0.89	0.87	681
	Macro average		0.63	0.62	0.62		0.64	0.61	0.62	910
	Weighted average		0.77	0.78	0.78		0.78	0.79	0.78	910

For the precision, recall, and F1 scores in the developing set, the random forest model achieved a macro average precision of 0.68, recall of 0.50, and F1 score of 0.54, with a weighted average precision of 0.77, recall of 0.79, and F1 score of 0.75. In comparison, the XGBoost model achieved a macro average precision of 0.73, recall of 0.53, and F1 score of 0.57, with a weighted average precision of 0.80, recall of 0.81, and F1 score of 0.77 (Table 3).

**Table 3:** Comparison of diagnostic efficacy between Random Forest and XGBoost

			RandomForest			XGBoost			
Dataset	Class		AUC	Obuchowski index	Accuracy	AUC	Obuchowski index	Accuracy	Delong test *p*-value (Z)
Developing set	0 (Granulomatous inflammation)		0.75	0.7599 (0.7361–0.7838)	0.79	0.79	0.8110 (0.7897–0.8303)	0.81	0.0029 (−2.975)
	1 (Benign tumors)		0.77			0.84			
	2 (Non-small cell lung cancer)		0.77			0.81			
Test set	0 (Granulomatous inflammation)		0.69	0.7193 (0.6567–0.7812)	0.80	0.76	0.8283 (0.7883–0.8650)	0.81	0.0029 (−2.975)
	1 (Benign tumors)		0.75			0.92			
	2 (Non-small cell lung cancer)		0.72			0.81			
Exvad set	0 (Granulomatous inflammation)		0.77	0.7932 (0.7572–0.8250)	0.78	0.79	0.8074 (0.7740–0.8387)	0.79	0.7560 (−0.311)
	1 (Benign tumors)		0.82			0.84			
	2 (Non-small cell lung cancer)		0.79			0.80			

Delong test: Random forest vs XGBoost

In the test set, the random forest model achieved AUC values of 0.69, 0.75, and 0.72 for classes 0, 1, and 2, respectively (Fig. 4b), with an Obuchowski index of 0.7193 (95% CI: 0.6567–0.7812), and an accuracy of 0.80. For the XGBoost model, the AUC values were 0.76, 0.92, and 0.81 for classes 0, 1, and 2, respectively (Fig. 4e), with an Obuchowski index of 0.8283 (95% CI: 0.7883–0.8650), and an accuracy of 0.81. The Delong test comparing the AUCs between the two models in the test set showed a *p*-value of 0.0029 and a Z score of −2.975, indicating a statistically significant difference in model performance (Table 2).

For the precision, recall, and F1 scores in the test set, the random forest model achieved a macro average precision of 0.67, recall of 0.56, and F1 score of 0.59, with a weighted average precision of 0.77, recall of 0.80, and F1 score of 0.78. In comparison, the XGBoost model achieved a macro average precision of 0.76, recall of 0.55, and F1 score of 0.61, with a weighted average precision of 0.80, recall of 0.81, and F1 score of 0.78 (Table 3).

In the Exvad set, the random forest model achieved AUC values of 0.77, 0.82, and 0.79 for classes 0, 1, and 2, respectively (Fig. 4c), with an Obuchowski index of 0.7932 (95% CI: 0.7572–0.8250), and an accuracy of 0.78. For the XGBoost model, the AUC values were 0.79, 0.84, and 0.80 for classes 0, 1, and 2, respectively (Fig. 4f), with an Obuchowski index of 0.8074 (95% CI: 0.7740–0.8387), and an accuracy of 0.79. The Delong test comparing the AUCs between the two models in the Exvad set showed a *p*-value of 0.7560 and a Z score of −0.311, indicating no statistically significant difference in model performance (Table 2).

For the precision, recall, and F1 scores in the Exvad set, the random forest model achieved a macro average precision of 0.63, recall of 0.62, and F1 score of 0.62, with a weighted average precision of 0.77, recall of 0.78, and F1 score of 0.78. In comparison, the XGBoost model achieved a macro average precision of 0.64, recall of 0.61, and F1 score of 0.62, with a weighted average precision of 0.78, recall of 0.79, and F1 score of 0.78 (Table 3).

Figure 4g, h displays the calibration curves of the two models, while Fig. 4i–k shows the DCA benefits of the two models across three datasets (Fig. 4).

### Interpretation of the model

**Fig. 5:**
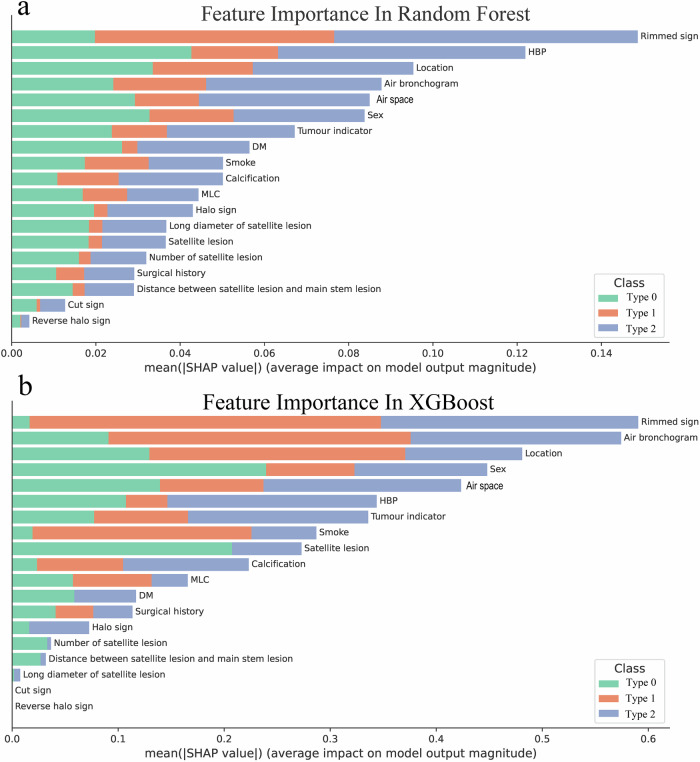
SHAP explanations for the random forest classifier (**a**) and the extreme gradient boosting (XGBoost) classifier (**b**). DM, diabetes mellitus; MLC, multiple lung comorbidity

In the random forest model, the five features with the highest importance, as determined by the SHAP values, are the rimmed sign, HBP, location, air bronchogram, and air space (Fig. 5a). Conversely, in the XGBoost model, the five features with the greatest importance are rimmed sign, air bronchograms, location, sex, and air space (Fig. 5b).

Individual Conditional Expectation (ICE) plots were constructed to illustrate the impact of critical features on the model’s predictions. Three distinct plots, each corresponding to one of the classification outcomes—granulomatous inflammation (Fig. S2), benign tumors (Fig. S3), and non-small cell lung cancer (NSCLC) (Fig. S4)—are presented. These plots elucidate the manner in which individual features affect model predictions across varying levels of feature values, thereby highlighting the variability in predictions for each class.

**Fig. 6:**
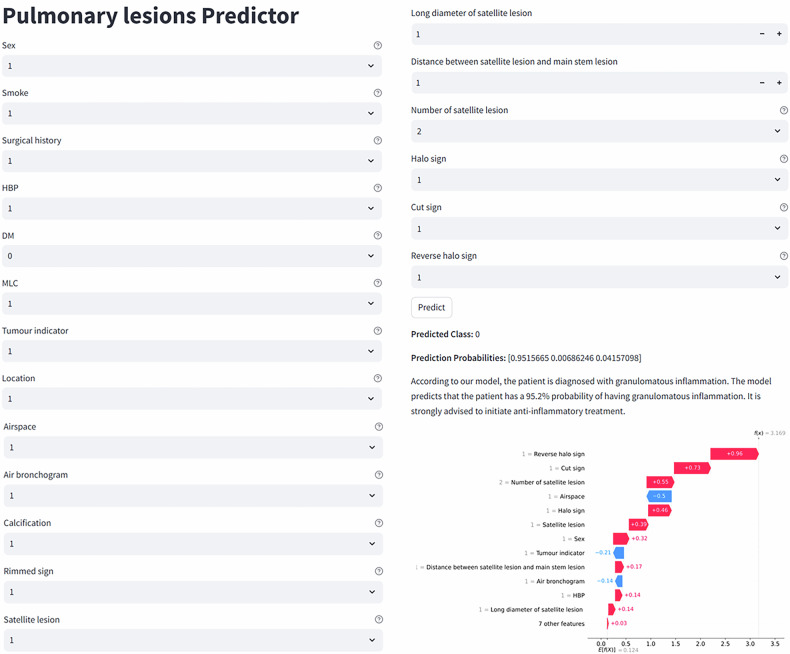
The predictive model based on the XGBoost algorithm was deployed online (https://yjdgejpxfeykfcbbjqzgx7.streamlit.app), with granulomatous inflammation coded as Class 0, benign tumors as Class 1, and non-small cell lung cancer as Class 2. HBP, high blood pressure; DM, diabetes mellitus; MLC, multiple lung comorbidity

The SHAP heatmap shows that the Rimmed sign exhibits a strong negative association with granulomatous inflammation (SHAP value = −0.1) but a slight positive contribution to NSCLC (SHAP value = 0.0032). The Air bronchogram has a pronounced negative impact on benign tumors (SHAP value = −0.057) and a moderate positive effect on NSCLC (SHAP value = 0.036). Features such as the cut sign and reverse halo sign display near-zero SHAP values across all classes, indicating minimal contribution to the model’s predictions (Fig. S5). Additionally, the online prediction website provides SHAP plots for individual patients, which can effectively assist radiologists in predicting the lesion type for each patient (Fig. 6).

## Discussion

To our knowledge, this is a retrospective, multicenter study aimed at exploring and establishing machine learning models to classify and differentiate pulmonary lesions. We identified predictive risk factors through univariate analysis and developed two predictive models.

Many studies have focused on binary classification predictions for pulmonary lesions. However, clinical conditions often present the possibility of multiclassification, and conventional binary classification models may struggle to address clinical issues accurately [17, 18]. Owing to their ability to handle complex and extensive data, machine learning techniques can construct precise predictive models by learning from large datasets. Integrating machine learning with clinical and imaging data can advance the development of clinical prediction models. Previous studies have shown that both the random forest and XGBoost models perform well in multiclass classification tasks [19–22]. Therefore, our study selected these two classical models for predictive analysis, model comparison, and interpretation. Utilizing the ROC curve, Obuchowski index, and calibration curves, our analysis demonstrates that the XGBoost model outperforms the random forest model in detection performance. Additionally, XGBoost exhibits a more robust fit, and the decision curve analysis (DCA) shows a greater benefit for XGBoost compared to random forest.

Owing to the lack of guidelines or consensus on feature selection for predictive models, the number of features to include remains elusive. Although numerous quantifiable features, such as radiomic features, have been developed, including many noncausal features, they may reduce the model’s predictive efficacy. Therefore, we used the Boruta algorithm to select important features, reducing data redundancy, and validated the results using RFE. After feature selection, the XGBoost model achieved efficient and stable performance on both the developing and test sets, with Obuchowski indices of 0.76 and 0.80 and accuracies of 0.79 and 0.77, respectively, indicating excellent multiclass classification performance.

Compared with traditional classification models, this study expanded the feature set by providing detailed descriptions of rimmed sign and satellite lesions. In the final model interpretation, certain features related to the rimmed sign and satellite lesion were found to play a significant role in the model’s predictions.

Machine learning has often been described as a ‘black box,’ with few studies explaining how prediction models are derived. This has led to difficulties in clinical application, which is another advantage of this research. We utilized the SHAP method to interpret the ‘black box’ of machine learning models [23]. The SHAP method provides global explanations that describe the overall function of the model. The study revealed that despite differences in SHAP-based feature importance between the two models, there were notable similarities in feature ranking and distribution. These findings indicate that different models similarly classify pulmonary diseases, offering valuable insights for clinical evaluation. Emphasis should be placed on features important in both models, such as rimmed sign, air bronchograms, location, air space, and tumor indications [24, 25].

We acknowledge several limitations of this study. First, radiomics models were not utilized to broaden the scope of our study. Furthermore, our research was concentrated on tertiary hospitals, leaving the efficacy of the model in primary and secondary healthcare settings unexplored and unvalidated. While our model demonstrates robust performance within tertiary hospital settings, its generalizability may be influenced by demographic homogeneity and selection bias in the study cohort. For instance, rural populations, ethnic minorities, and early-stage disease cases are underrepresented. Future studies should prioritize multicenter validation across diverse geographic and socioeconomic contexts, coupled with bias-mitigation strategies such as data harmonization and fairness-aware machine learning. These efforts will ensure the equitable deployment of AI tools in real-world healthcare systems. Additionally, we did not perform specific classifications of granulomatous inflammation. Given that the etiology of granulomatous inflammation can be bacterial, fungal, or viral, further investigation into lesions caused by different etiologies is warranted. Future work should integrate radiomics, deep learning architectures, and multicenter validation across diverse populations, alongside bias mitigation to ensure equitable AI deployment.

Our study can be summarized in two key points. First, we developed a three-class lung disease prediction model and identified key risk factors for disease prediction. Second, refined indicators such as the rimmed sign and satellite lesion hold significant value in the differential diagnosis of lung diseases.

## Supplementary information

ELECTRONIC SUPPLEMENTARY MATERIAL

## Data Availability

If needed, please contact the corresponding author.

## Abbreviations

AUC: Area under the curve
CT: X-ray computed tomography
DCA: Decision curve analysis
DM: Diabetes mellitus
HBP: High blood pressure
HU: Hounsfield unit
ICE: Individual conditional expectation
LD: Long distance
mGGO: Mixed ground-glass opacity
MLC: Multiple lung comorbidity
RF: Random forest
ROC: Receiver operating characteristic
SD: Short distance
SHAP: Shapley additive explanations
XGBoost: Extreme gradient boosting
