# Supplementary material for: Development, deployment, and feature interpretability of a three-class prediction model for pulmonary diseases
Source: Insights Imaging. 2025 Jun 26;16:133. doi: 10.1186/s13244-025-02020-7 (PMC12202249; doi:10.1186/s13244-025-02020-7)

Development, deployment, and feature interpretability of a three-class prediction model for pulmonary diseases

ELECTRONIC SUPPLEMENTARY MATERIAL

TableS1: Comparison of differences between the two provinces

Characteristic	Total (n = 3030)	1 (n = 2120)	2 (n = 910)	Statistic	*P*
Age, Mean ± SD	59.62 ± 11.24	59.30 ± 10.97	60.37 ± 11.84	t=-2.33	**0.020**
LD, Mean ± SD	19.16 ± 10.72	18.18 ± 10.14	21.46 ± 11.67	t=-7.37	**<.001**
SD, Mean ± SD	14.31 ± 8.09	13.55 ± 7.62	16.09 ± 8.85	t=-7.55	**<.001**
CTmax, Mean ± SD	88.44 ± 186.32	77.75 ± 199.23	113.35 ± 149.21	t=-5.42	**<.001**
CTmin, Mean ± SD	-186.74 ± 227.11	-198.33 ± 236.22	-159.74 ± 201.86	t=-4.58	**<.001**
CTmean, Mean ± SD	-44.54 ± 184.01	-57.89 ± 197.70	-13.42 ± 142.59	t=-6.96	**<.001**
CTsd, Mean ± SD	78.58 ± 50.39	82.41 ± 50.75	69.66 ± 48.42	t=6.55	**<.001**
Long diameter of satellite lesion, Mean ± SD	0.22 ± 1.32	0.11 ± 0.92	0.47 ± 1.94	t=-5.35	**<.001**
Distance between satellite lesion and main stem lesion, Mean ± SD	0.31 ± 1.82	0.18 ± 1.40	0.63 ± 2.51	t=-5.06	**<.001**
**Clinical Characteristics**					
Sex, n(%)				χ^2=3.25	0.072
Female	1647 (54.36)	1175 (55.42)	472 (51.87)		
Male	1383 (45.64)	945 (44.58)	438 (48.13)		
Pathology Type, n(%)				χ^2=0.00	0.999
Granulomatous inflammation	529 (17.46)	370 (17.45)	159 (17.47)		
Benign tumors	232 (7.66)	162 (7.64)	70 (7.69)		
Non-small cell lung cancer	2269 (74.88)	1588 (74.91)	681 (74.84)		
Smoke, n(%)				χ^2=17.47	**<.001**
NO	2546 (84.03)	1820 (85.85)	726 (79.78)		
Yes	484 (15.97)	300 (14.15)	184 (20.22)		
Surgical history, n(%)				χ^2=0.59	0.443
NO	2668 (88.05)	1873 (88.35)	795 (87.36)		
Yes	362 (11.95)	247 (11.65)	115 (12.64)		

Characteristic	Total (n = 3030)	1 (n = 2120)	2 (n = 910)	Statistic	*P*
HBP, n(%)				χ²=370.81	**<.001**
NO	2742 (90.50)	2061 (97.22)	681 (74.84)		
Yes	288 (9.50)	59 (2.78)	229 (25.16)		
DM, n(%)				χ²=107.21	**<.001**
NO	2904 (95.84)	2084 (98.30)	820 (90.11)		
Yes	126 (4.16)	36 (1.70)	90 (9.89)		
Emphysema or Bullae of lung, n(%)				χ²=23.19	**<.001**
NO	2501 (82.54)	1796 (84.72)	705 (77.47)		
Yes	529 (17.46)	324 (15.28)	205 (22.53)		
HVP, n(%)				χ²=4.94	**0.026**
NO	2652 (87.52)	1837 (86.65)	815 (89.56)		
Yes	378 (12.48)	283 (13.35)	95 (10.44)		
ID, n(%)				χ²=41.09	**<.001**
NO	2989 (98.65)	2110 (99.53)	879 (96.59)		
Yes	41 (1.35)	10 (0.47)	31 (3.41)		
Bronchiectasis, n(%)				χ²=9.17	**0.002**
NO	2999 (98.98)	2106 (99.34)	893 (98.13)		
Yes	31 (1.02)	14 (0.66)	17 (1.87)		
MLC, n(%)				χ²=8.75	**0.003**
NO	2931 (96.73)	2064 (97.36)	867 (95.27)		
Yes	99 (3.27)	56 (2.64)	43 (4.73)		
Tumour indicator, n(%)				χ²=1.85	0.174
NO	2233 (73.75)	1577 (74.46)	656 (72.09)		
Yes	795 (26.25)	541 (25.54)	254 (27.91)		
**Radiological Characteristics**					
Location, n(%)				χ²=12.66	**0.013**
Right upper lobe	930 (30.69)	677 (31.93)	253 (27.80)		
Right middle lobe	241 (7.95)	177 (8.35)	64 (7.03)		
Right lower lobe	632 (20.86)	446 (21.04)	186 (20.44)		
Left upper lobe	714 (23.56)	488 (23.02)	226 (24.84)		
Left lower lobe	513 (16.93)	332 (15.66)	181 (19.89)		
Morphology, n(%)				χ²=22.27	<.001
NO	201 (6.63)	111 (5.24)	90 (9.89)		
Yes	2829 (93.37)	2009 (94.76)	820 (90.11)		

Characteristic	Total (n = 3030)	1 (n = 2120)	2 (n = 910)	Statistic	*P*
Lobulation, n(%)				-	**<.001**
NO	1210 (39.93)	874 (41.23)	336 (36.92)		
Yes	1820 (60.07)	1246 (58.77)	574 (63.08)		
Spiculation, n(%)				χ^2=18.50	<.001
NO	1996 (65.87)	1448 (68.30)	548 (60.22)		
Yes	1034 (34.13)	672 (31.70)	362 (39.78)		
Airspace, n(%)				χ^2=5.97	0.015
NO	2505 (82.67)	1776 (83.77)	729 (80.11)		
Yes	525 (17.33)	344 (16.23)	181 (19.89)		
Air bronchogram, n(%)				χ^2=23.86	<.001
NO	2440 (80.53)	1756 (82.83)	684 (75.16)		
Yes	590 (19.47)	364 (17.17)	226 (24.84)		
Pleural tags, n(%)				χ^2=57.20	**<.001**
Type 0	1019 (33.63)	751 (35.42)	268 (29.45)		
Type Ⅰ	1208 (39.87)	881 (41.56)	327 (35.93)		
Type Ⅱ	360 (11.88)	242 (11.42)	118 (12.97)		
Type Ⅲ	142 (4.69)	77 (3.63)	65 (7.14)		
Type Ⅳ	301 (9.93)	169 (7.97)	132 (14.51)		
Calcification, n(%)				χ^2=0.30	0.584
NO	2922 (96.44)	2047 (96.56)	875 (96.15)		
Yes	108 (3.56)	73 (3.44)	35 (3.85)		
Pleural effusion, n(%)				χ^2=4.65	**0.031**
NO	3011 (99.37)	2111 (99.58)	900 (98.90)		
Yes	19 (0.63)	9 (0.42)	10 (1.10)		
Rimmed sign, n(%)				χ^2=78.50	**<.001**
NO	2707 (89.34)	1963 (92.59)	744 (81.76)		
Yes	323 (10.66)	157 (7.41)	166 (18.24)		
Rimmed sign$^{\alpha}$, n(%)				χ^2=95.21	**<.001**
L=0%	2708 (89.37)	1963 (92.59)	745 (81.87)		
L<25%	17 (0.56)	5 (0.24)	12 (1.32)		
25%≤L<50%	37 (1.22)	10 (0.47)	27 (2.97)		
50%≤L<75%	57 (1.88)	27 (1.27)	30 (3.30)		
75%≤L<100%	90 (2.97)	43 (2.03)	47 (5.16)		
L=100%	121 (3.99)	72 (3.40)	49 (5.38)		

Characteristic	Total (n = 3030)	1 (n = 2120)	2 (n = 910)	Statistic	*P*
Rimmed sign$^{\beta}$, n(%)				χ^2=96.58	**<.001**
N=0	2708 (89.37)	1963 (92.59)	745 (81.87)		
N=1	187 (6.17)	109 (5.14)	78 (8.57)		
N=2	56 (1.85)	21 (0.99)	35 (3.85)		
N≥3	79 (2.61)	27 (1.27)	52 (5.71)		
Satellite lesion, n(%)				χ^2=59.38	**<.001**
No	2901 (95.74)	2069 (97.59)	832 (91.43)		
Yes	129 (4.26)	51 (2.41)	78 (8.57)		
Number of satellite lesion, n(%)				χ^2=70.37	**<.001**
N=0	2901 (95.74)	2069 (97.59)	832 (91.43)		
N=1	49 (1.62)	13 (0.61)	36 (3.96)		
N=2	14 (0.46)	10 (0.47)	4 (0.44)		
N≥3	66 (2.18)	28 (1.32)	38 (4.18)		
Halo sign, n(%)				χ^2=50.03	**<.001**
No	2945 (97.19)	2090 (98.58)	855 (93.96)		
Yes	85 (2.81)	30 (1.42)	55 (6.04)		
Cut sign, n(%)				χ^2=14.30	**<.001**
No	3001 (99.04)	2109 (99.48)	892 (98.02)		
Yes	29 (0.96)	11 (0.52)	18 (1.98)		
Reverse halo sign, n(%)				χ^2=0.02	0.882
No	3021 (99.70)	2113 (99.67)	908 (99.78)		
Yes	9 (0.30)	7 (0.33)	2 (0.22)		
Rimmed sign$^{\alpha}$, n(%)				χ^2=95.21	**<.001**
L=0%	2708 (89.37)	1963 (92.59)	745 (81.87)		
L<25%	17 (0.56)	5 (0.24)	12 (1.32)		
25%≤L<50%	37 (1.22)	10 (0.47)	27 (2.97)		
50%≤L<75%	57 (1.88)	27 (1.27)	30 (3.30)		
75%≤L<100%	90 (2.97)	43 (2.03)	47 (5.16)		
L=100%	121 (3.99)	72 (3.40)	49 (5.38)		

NOTE. t: t-test, χ^2: Chi-square test, -: Fisher exact. SD: standard deviation

TableS2: Kappa and ICC Values for Diagnostic Consistency Assessment

Feature	Kappa(1,1)	Kappa(1,2)	ICC(1,1)	ICC(1,1) CI95%	ICC(1,2)	ICC(1,2) CI95%
Emphysema or Bullae of lung	0.89	0.89				
HVP	1.00	1.00				
ID	1.00	1.00				
Bronchiectasis	1.00	1.00				
MLC	1.00	1.00				
Tumour indicator	1.00	1.00				
mGGO	0.89	0.80				
Location	1.00	1.00				
Morphology	1.00	1.00				
Lobulation	0.84	0.82				
Spiculation	0.93	0.91				
Airspace	1.00	1.00				
Air bronchogram	1.00	1.00				
Pleural tags	0.84	0.84				
LD			0.95	[0.90-1.00]	0.92	[0.88-0.95]
SD			0.91	[0.85-0.97]	0.90	[0.84-0.95]
CTmax			0.89	[0.88-0.91]	0.88	[0.87-0.90]
CTmin			0.85	[0.78-0.90]	0.82	[0.75-0.89]
CTmean			0.86	[0.82-0.90]	0.86	[0.82-0.89]
CTsd			0.88	[0.80-0.96]	0.83	[0.77-0.88]
Calcification	0.98	0.95				
Pleural effusion	1.00	1.00				
Rimmed sign	0.87	0.87				
Rimmed sign$^{\alpha}$	0.88	0.88				
Rimmed sign$^{\beta}$	0.88	0.88				
Satellite lesion	0.95	0.92				
Long diameter of satellite lesion			0.95	[0.92-0.99]	0.96	[0.93-0.99]
Distance between satellite lesion and main stem lesion			0.96	[0.92-0.99]	0.97	[0.94-0.99]
Number of satellite lesion	1.00	1.00				

Halo sign	1.00	1.00
Cut sign	0.95	0.88
Reverse halo sign	1.00	0.87

Note. ICC > 0.75: Good consistency, Kappa > 0.6: Substantial agreement, Kappa > 0.8: Almost perfect agreement. Kappa (1,1) measures agreement between two raters on a categorical outcome, adjusting for chance. Kappa (1,2) assesses agreement between two raters on a categorical outcome, allowing for multiple ratings per subject. ICC (1,1) evaluates the consistency of a single rater's measurements against overall variability. ICC (1,2) measures the consistency of a single rater's measurements against the average from multiple raters.

TableS3: Model Performance with Different Sampling Approaches

Sampler	Model	Train_ACC	Train_F1	Train_AUC	Test_ACC	Test_F1	Test_AUC
None	KNN	0.752	0.697	0.620	0.797	0.763	0.715
None	LR	0.775	0.730	0.731	0.802	0.770	0.737
None	MLP	0.762	0.721	0.706	0.785	0.768	0.715
None	RF	0.741	0.699	0.706	0.790	0.775	0.776
None	SVM	0.759	0.712	0.710	0.811	0.785	0.782
None	XGB	0.749	0.712	0.727	0.781	0.762	0.773
SMOTE	KNN	0.624	0.621	0.759	0.748	0.743	0.712
SMOTE	LR	0.538	0.535	0.750	0.526	0.567	0.732
SMOTE	MLP	0.663	0.658	0.847	0.514	0.557	0.695
SMOTE	RF	0.727	0.725	0.892	0.682	0.697	0.753
SMOTE	SVM	0.590	0.586	0.784	0.604	0.636	0.721
SMOTE	XGB	0.725	0.721	0.893	0.677	0.694	0.745
BorderSMOTE	KNN	0.460	0.407	0.610	0.792	0.775	0.720
BorderSMOTE	LR	0.541	0.543	0.745	0.495	0.537	0.687
BorderSMOTE	MLP	0.668	0.667	0.844	0.538	0.578	0.711
BorderSMOTE	RF	0.695	0.694	0.870	0.533	0.573	0.737
BorderSMOTE	SVM	0.628	0.624	0.803	0.446	0.488	0.652
BorderSMOTE	XGBoost	0.688	0.688	0.862	0.564	0.600	0.735
SVMSMOTE	KNN	0.660	0.657	0.770	0.788	0.770	0.706
SVMSMOTE	LR	0.673	0.672	0.864	0.689	0.698	0.687
SVMSMOTE	MLP	0.721	0.718	0.906	0.670	0.686	0.712

SVMSMOTE	RF	0.773	0.772	0.933	0.639	0.663	0.747
SVMSMOTE	SVM	0.699	0.701	0.869	0.691	0.703	0.734
SVMSMOTE	XGBoost	0.768	0.766	0.927	0.653	0.674	0.739
RandomOver	KNN	0.592	0.579	0.733	0.778	0.770	0.713
RandomOver	LR	0.536	0.533	0.751	0.573	0.615	0.743
RandomOver	MLP	0.699	0.696	0.884	0.599	0.635	0.723
RandomOver	RF	0.738	0.736	0.909	0.590	0.625	0.744
RandomOver	SVM	0.637	0.634	0.819	0.634	0.664	0.748
RandomOver	XGBoost	0.725	0.724	0.900	0.637	0.665	0.751
RandomUnder	KNN	0.569	0.552	0.781	0.304	0.294	0.676
RandomUnder	LR	0.526	0.525	0.753	0.502	0.547	0.762
RandomUnder	MLP	0.733	0.733	0.910	0.533	0.578	0.777
RandomUnder	RF	0.764	0.764	0.930	0.512	0.556	0.771
RandomUnder	SVM	0.603	0.603	0.794	0.514	0.561	0.743
RandomUnder	XGBoost	0.762	0.761	0.925	0.571	0.612	0.789

NOTE. LR：Logistic Regression, SVM：Support Vector Machine, KNN：k-Nearest Neighbors, RF：Random Forest, XGB：XGBoost, MLP：Multi-layer Perceptron, SMOTE: Synthetic Minority Oversampling Technique, ACC: Accuracy, F1: F1 Score, AUC: Area Under the Curve, Border SMOTE: Borderline Synthetic Minority Over-sampling Technique, SVMSMOTE: Support Vector Machine-based Synthetic Minority Over-sampling Technique, RandomOver: Random Over-sampling, RandomUnder: Random Under-Sampling.

TableS4: Average performance metrics of machine learning models across different sampling techniques.

Sampler	Train_ACC	Train_F1	Train_AUC	Test_ACC	Test_F1	Test_AUC
RandomUnder	0.659	0.657	0.849	0.489	0.525	0.753
None	0.756	0.712	0.700	0.794	0.771	0.750
RandomOver	0.655	0.650	0.833	0.635	0.662	0.737
SVMSMOTE	0.716	0.714	0.878	0.688	0.699	0.721
SMOTE	0.644	0.641	0.821	0.625	0.649	0.726
BorderSMOTE	0.613	0.604	0.789	0.561	0.592	0.707

TableS5: Average performance comparison of different machine learning algorithms under various sampling methods.

Model	Train_ACC	Train_F1	Train_AUC	Test_ACC	Test_F1	Test_AUC
KNN	0.610	0.585	0.712	0.701	0.686	0.707
LR	0.598	0.590	0.766	0.598	0.622	0.725
MLP	0.708	0.699	0.849	0.607	0.634	0.722
RF	0.740	0.732	0.873	0.624	0.648	0.754
SVM	0.653	0.643	0.796	0.617	0.640	0.730
XGBoost	0.736	0.729	0.872	0.647	0.668	0.755

NOTE. LR：Logistic Regression, SVM：Support Vector Machine, KNN：k-Nearest Neighbors, RF：Random Forest, XGB：XGBoost, MLP：Multi-layer Perceptron, SMOTE: Synthetic Minority Oversampling Technique, ACC: Accuracy, F1: F1 Score, AUC: Area Under the Curve, Border SMOTE: Borderline Synthetic Minority Over-sampling Technique, SVMSMOTE: Support Vector Machine-based Synthetic Minority Over-sampling Technique, RandomOver: Random Over-sampling, RandomUnder: Random Under-Sampling.

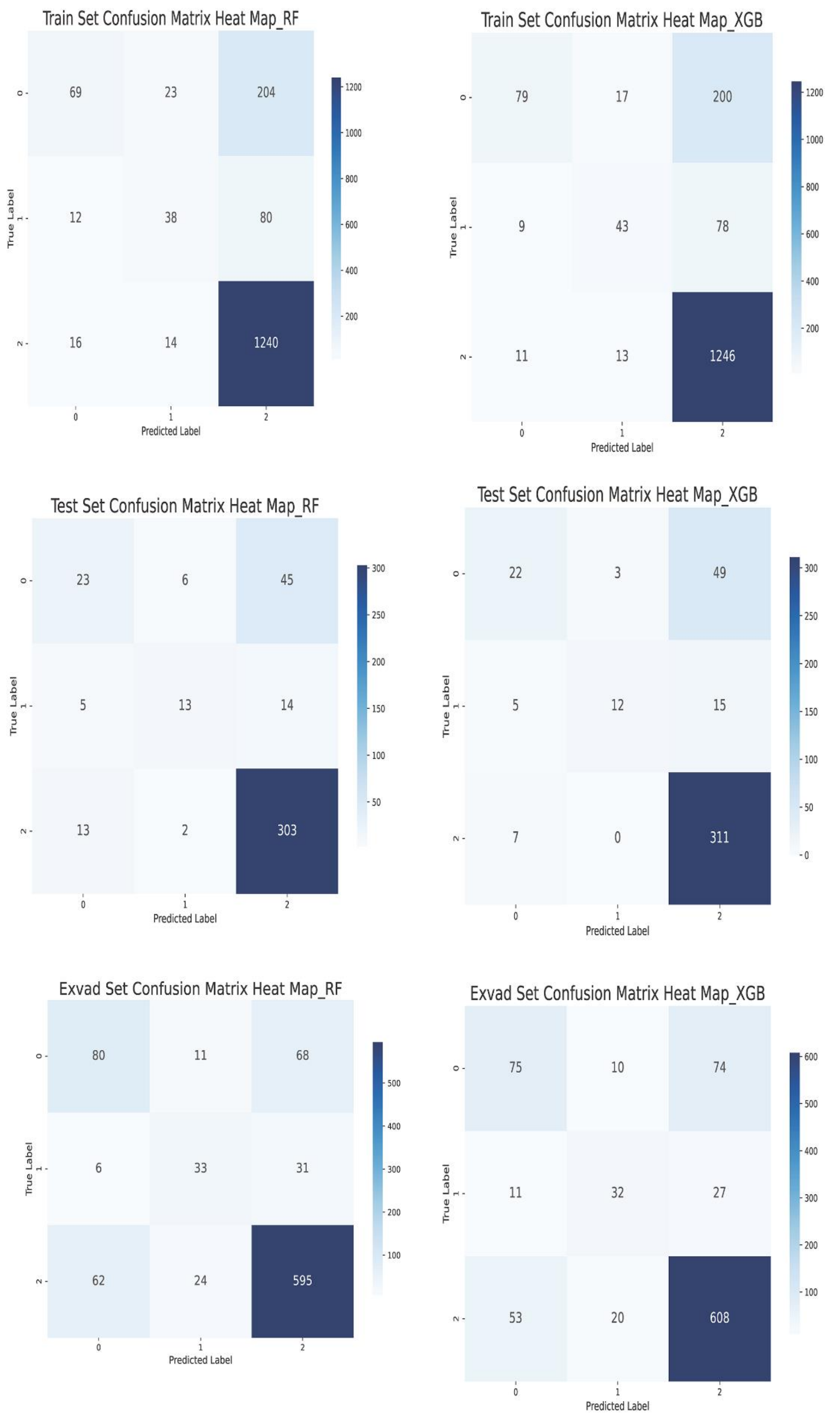

Figure S1. shows the confusion matrices of the Random Forest model and XGBoost model, respectively.

Figure S2. This is an ICE curve for differentiating Granulomatous inflammation from non-Granulomatous inflammation diseases. HBP, high blood pressure; DM, diabetes mellitus; MLC, multiple lung comorbidity. The ICE curve illustrates the relationship between the feature variable and the predicted outcome. A slope greater than 0 indicates a positive correlation, a slope less than 0 indicates a negative correlation, and any bending of the curve suggests a nonlinear relationship.

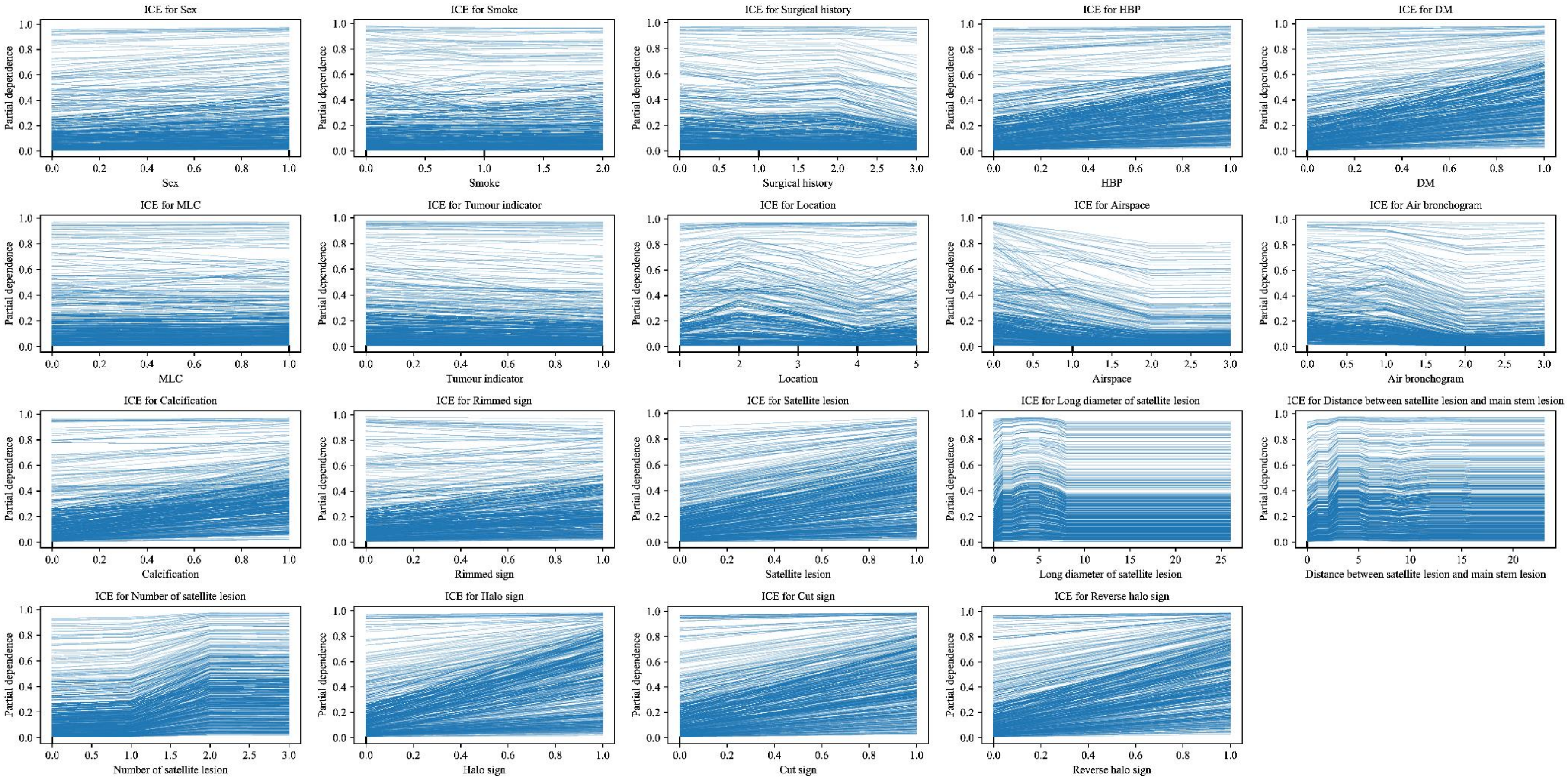

Figure S3. This is an ICE curve for differentiating Benign tumors from non-Benign tumors diseases. HBP, high blood pressure; DM, diabetes mellitus; MLC, multiple lung comorbidity. The ICE curve illustrates the relationship between the feature variable and the predicted outcome. A slope greater than 0 indicates a positive correlation, a slope less than 0 indicates a negative correlation, and any bending of the curve suggests a nonlinear relationship.

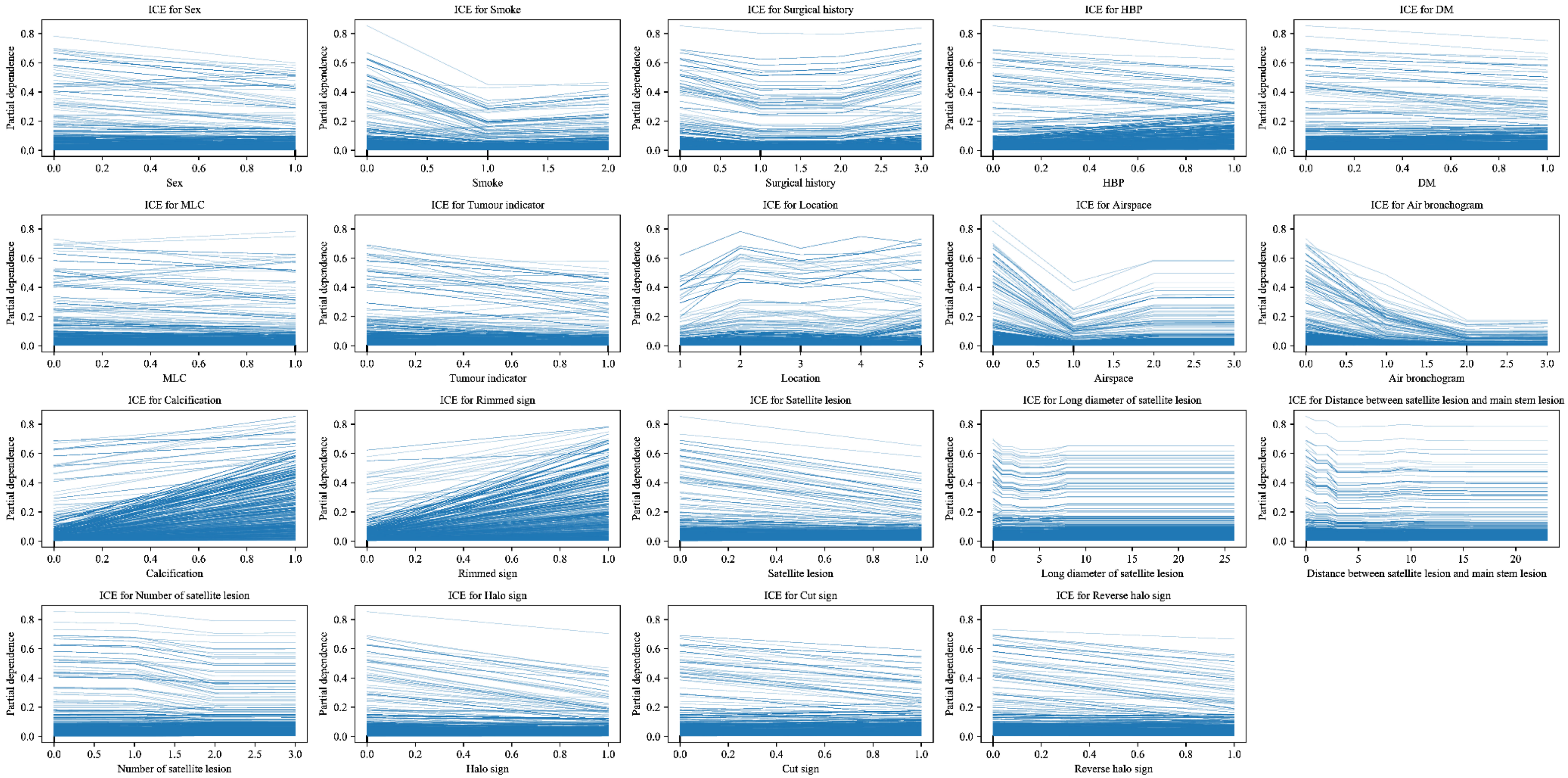

Figure S4. This is an ICE curve for differentiating Non-small cell lung cancer from non-Non-small cell lung cancer diseases. HBP, high blood pressure; DM, diabetes mellitus; MLC, multiple lung comorbidity. The ICE curve illustrates the relationship between the feature variable and the predicted outcome. A slope greater than 0 indicates a positive correlation, a slope less than 0 indicates a negative correlation, and any bending of the curve suggests a nonlinear relationship.

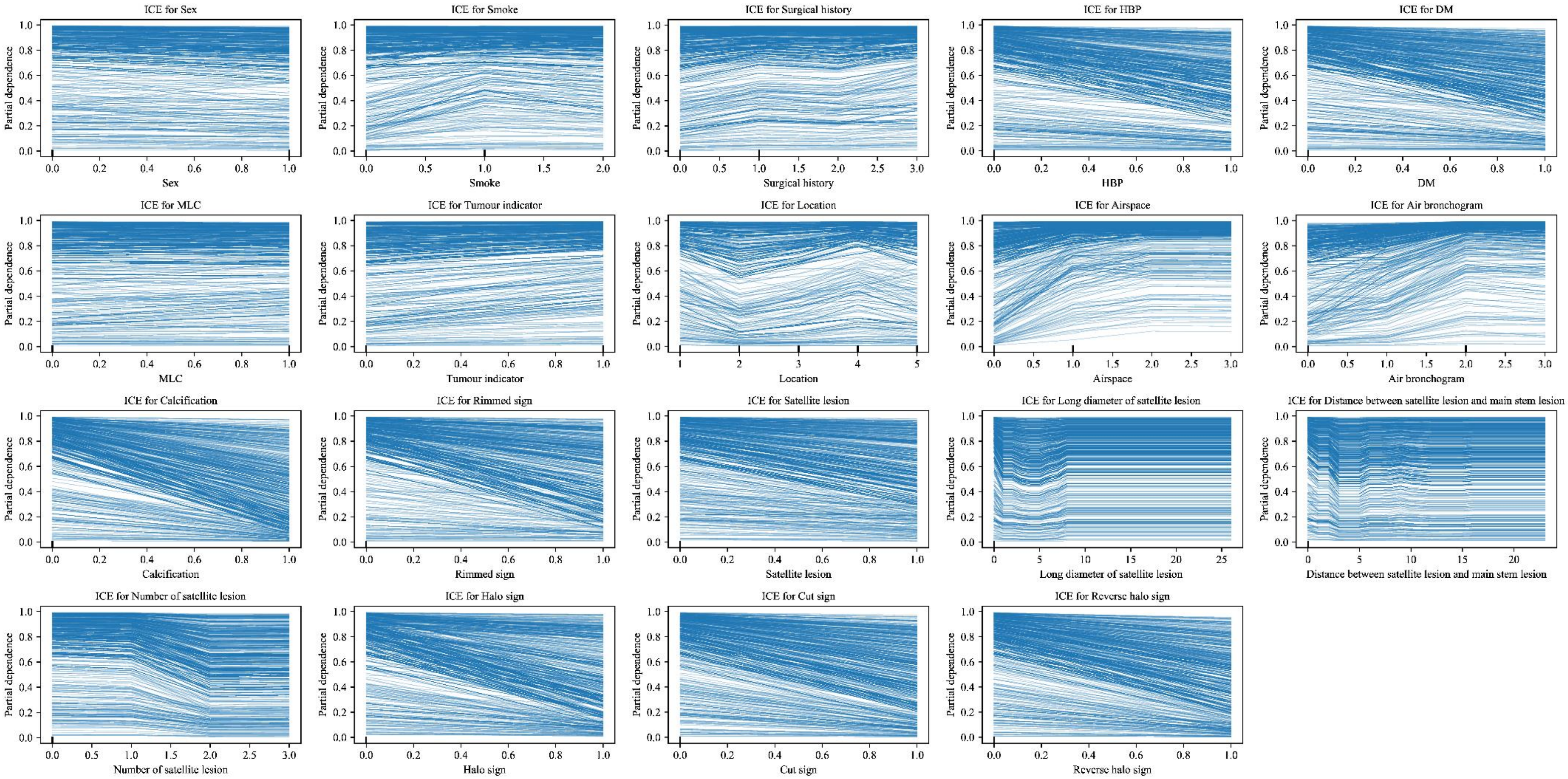

Figure S5. SHAP heatmap of the XGBoost. HBP, high blood pressure; DM, diabetes mellitus; MLC, multiple lung comorbidity.

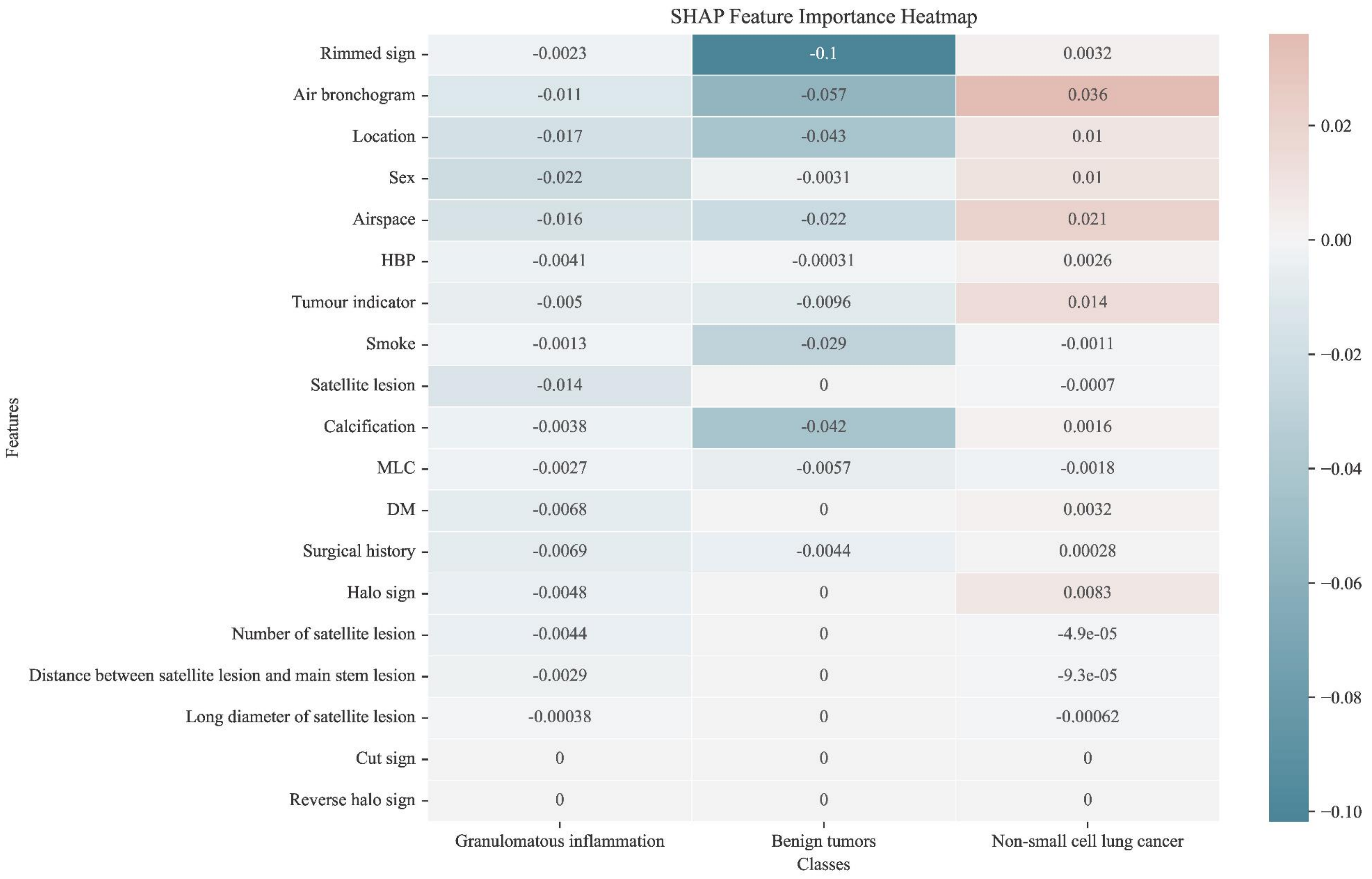

Supplement: Supplementary file 1 — ELECTRONIC SUPPLEMENTARY MATERIAL [file 13244_2025_2020_MOESM1_ESM.pdf]
